# Metatranscriptomic Analysis of *Pycnopodia helianthoides* (Asteroidea) Affected by Sea Star Wasting Disease

**DOI:** 10.1371/journal.pone.0128150

**Published:** 2015-05-28

**Authors:** Brent M. Gudenkauf, Ian Hewson

**Affiliations:** Department of Microbiology, Cornell University, Ithaca, New York, United States of America; USGS National Wildlife Health Center, UNITED STATES

## Abstract

Sea star wasting disease (SSWD) describes a suite of symptoms reported in asteroids of the North American Pacific Coast. We performed a metatranscriptomic survey of asymptomatic and symptomatic sunflower star (*Pycnopodia helianthoides*) body wall tissues to understand holobiont gene expression in tissues affected by SSWD. Metatranscriptomes were highly variable between replicate libraries, and most differentially expressed genes represented either transcripts of associated microorganisms (particularly *Pseudomonas *and *Vibrio *relatives) or low-level echinoderm transcripts of unknown function. However, the pattern of annotated host functional genes reflects enhanced apoptotic and tissue degradation processes and decreased energy metabolism, while signalling of death-related proteins was greater in asymptomatic and symptomatic tissues. Our results suggest that the body wall tissues of SSWD-affected asteroids may undergo structural changes during disease progression, and that they are stimulated to undergo autocatalytic cell death processes.

## Introduction

Sea stars (Echinodermata: Asteroidea) are crucial components of coastal marine ecosystems, with some considered keystone species due to their disproportionately large influence on benthic community structure relative to their abundance [1]. Echinoderms periodically experience massive mortality, often resulting in large ecosystem changes. For example, a mass mortality of the herbivorous urchin *Diadema antillarum* in Caribbean Reefs in the early 1980s [2] dramatically altered the composition of coral reef communities [3]; *Diadema antillarum* has still not regained its pre-epizootic abundance[4]. In Nova Scotia, mass mortalities of the urchin *Strongylocentrotus droebachiensis* shifted communities from barrens to lush kelp beds [5]. Hence, echinoderm disease has the potential to reshape benthic invertebrate communities over short to long time scales.

Sea star wasting disease (SSWD) describes a suite of objective clinical signs observed in asteroids, beginning with behavioral changes including lethargy and curling of limbs, followed by development of lesions prior to arm autotomy, loss of animal turgor (deflation), and finally animal death. Previous episodes of asteroid wasting have been limited to single species in restricted ranges. SSWD was reported in Southern California in 1978 [6], 1982–1983 [7] and 1997 [7], and in British Columbia in 2008 [8]. Wasting-like signs were also observed in asteroids in the South Pacific in 1984, 1986 [9] and 1999 [10]. Since June 2013, 20 species of asteroid, spanning much of the asteroid phylogeny, and from Alaska to Baja California have died from SSWD, making it the largest marine epizootic observed yet [11]. Sea star wasting disease is caused by a virus-sized organism, and the most promising candidate of viruses is the sea star associated densovirus (SSaDV) [11]. Densoviruses have previously been observed in urchins (echinoids) [12] and SSaDV was detected in both ophiuroids, echinoids and asteroids, suggesting densoviruses may be a common constituent of echinoderm holobionts. Like all marine invertebrates, echinoderms exist in a medium containing abundant heterotrophic bacteria (~ 10^6^ cells ml^-1^) that consume labile organic matter, including metazoan exudates. Echinoderms also bear water vascular systems which draw water through a sieve plate/madreporite [13]. This sieve plate permits bacteria-sized particles to pass into the water vascular system. The echinoderm holobiont therefore comprises the animal itself along with bacteria and other microorganisms.

The purpose of this study is to compare the gene transcription profile of symptomatic and asymptomatic sunflower stars (*Pycnopodia helianthoides*) affected by SSWD. Metatranscriptomes (comprising host + associated microorganisms) were prepared from body wall tissue extracts from symptomatic and asymptomatic asteroids during the onset of SSWD in October 2013. Our results suggest that body wall tissues of SSWD-affected asteroids experience enhanced tissue degradation, matrix remodeling, and decreased metabolic activity compared to asymptomatic tissues. Surprisingly, immunity-related genes are not more expressed in symptomatic and asymptomatic tissues.

## Materials and Methods

### Sample collection

The SSWD epidemic has affected asteroids since at least June 2013, with early reports from the Olympic National Park, WA, and shortly thereafter reports of mass mortality of asteroids in the northern Salish Sea (August 2013) [11]. By early October 2013, SSWD was reported in the southern Puget Sound. Asteroids at the Seattle Aquarium began to display SSWD signs by mid October 2013. On 26 October 2013, 2 visually asymptomatic and 2 SSWD-affected (i.e. symptomatic) sunflower stars (*Pycnopodia helianthoides*) were collected from the Seattle Aquarium. Samples were frozen at -80°C and shipped to the laboratory at Cornell University for further analysis.

### Metatranscriptome Preparation

Samples for metatranscriptomic analysis were thawed and small tissue samples from the body wall along a ray were excised and placed into sterile microcentrifuge tubes. RNA was extracted from samples using the ZR RNA Mini Isolation Kit (Zymo Research), and co-extracted DNA was removed using the DNA-free RNA kit (Zymo Research). RNA was then amplified using the Transplex WTA kit (Sigma Aldrich) following manufacturer’s recommendations and sequenced using 2 x 250 bp Illumina MiSeq chemistry at the Cornell University Biotechnology Resource Center. Each metatranscriptome was run on 1/12^th^ of a lane (lanes shared with un-related samples). All sequences have been deposited at GenBank under accessions SAMN0286752-SAMN0286755.

Sequences were imported into the CLC Genomics Workbench 4.0 and trimmed for quality (N<2 ambiguous bases) and size (sequences larger and smaller than 250 bp were discarded). A global assembly of all 4 metatranscriptomes was performed to provide longer contiguous sequences which afford greater confidence in annotation. The assembly was conducted with a minimum of 0.5 overlap and 0.8 identity. Following assembly, reads in each library were recruited separately to the globally assembled contigs using the same parameters as assembly. The percentage of reads associated with each contig was corrected for total library size.

We restricted annotation to contiguous sequences which either represented a large proportion of total reads across all four libraries, or those that were more than 10-fold and statistically significantly different (p< 0.05, Student’s *t*-test) between asymptomatic and symptomatic tissues. To establish contiguous sequences representing a large proportion of reads, the mean value was caluclated across all four libraries and corrected for contig size (since longer contigs may recruit more reads) by dividing percentage reads by contig length. Contigs in each category were annotated by BLASTx comparison against the non-redundant (nr) database at NCBI, with an expect value cut-off of 0.001.

## Results and Discussion

In this study, we compared metatranscriptomes between asymptomatic and animals showing signs of SSWD in an attempt to elucidate highly differentially represented genes which may give clues about how SSWD affects asteroid physiology. Some asymptomatic sea stars may have been in preclinical stages of the disease, as they lived alongside symptomatic stars. Like all marine invertebrates and especially echinoderms, asteroids bear associated microorganisms on their surfaces and within their water vascular systems. Our transcriptomes of host tissues which do not select for eukaryotic or prokaryotic transcripts represent metatranscriptomes of the echinoderm holobiont.

Metatranscriptome libraries each comprised 0.3–1.1 x 10^6^ reads per library (Table 1). Of 3.6 million sequence reads, 3.1 x 10^6^ recruited to 2.7 x 10^5^ contiguous sequences. 70–93% of reads in individual libraries recruited to globally assembled contigs. Of globally assembled contigs, ORFs on only 4.7 x 10^4^ contigs (17.7%) were homologous with proteins in the non-redundant database. Bacteria comprised the greatest length-normalized proportion of read annotations (54% of reads), while eukaryotic (37%) and viral (2%) annotations were fewer.

**Table 1:** Asteroid holobiont metatranscriptome charactertics and assembly statistics for global analysis.

Metatranscriptome Name	T1	T2	T3	T4	Global Assembly
**S/A**	A	A	S	S	
**Date**	10/26/2013	10/26/2013	10/26/2013	10/26/2013	
**Total # Reads**	1,104,495	307,895	1,060,875	1,079,434	3,552,699
**# Reads Recruited to Contigs**	1,031,508	261,562	971,675	758,362	3,119,777
**# Contigs**	-	-	-	-	265,887

All samples were from *Pycnopodia helianthoides* collected at the Seattle Aquarium on 10/26/13. A = Asymptomatic, S = Symptomatic.

**Fig 1:**
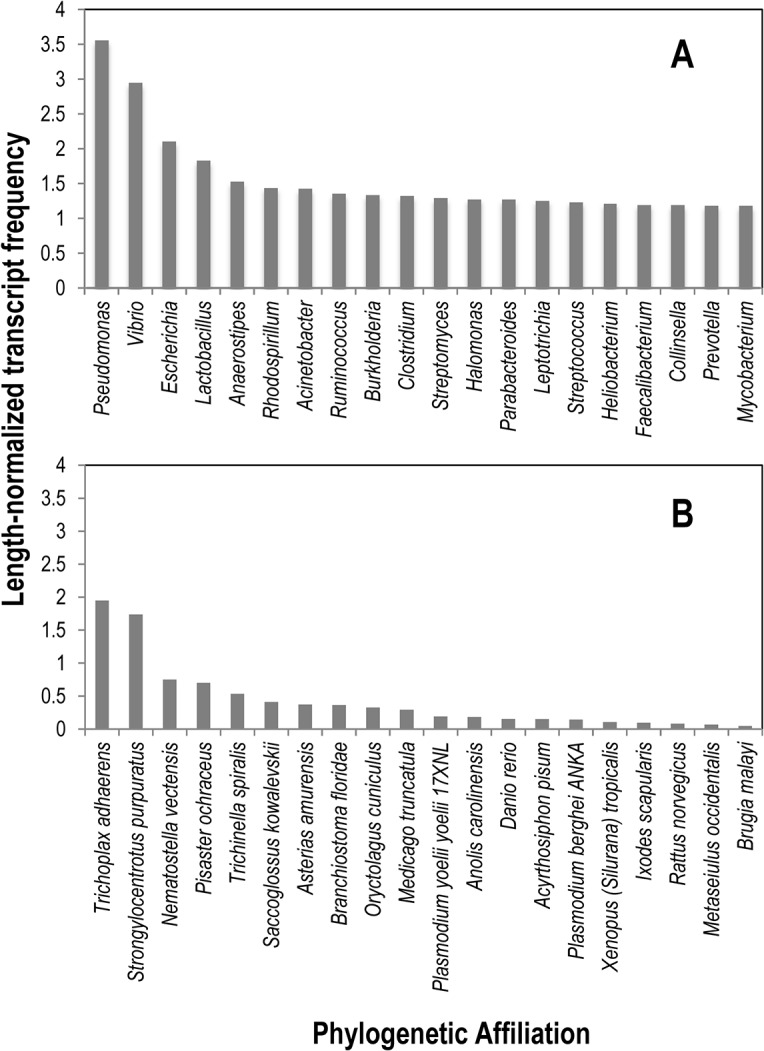
Twenty most abundant bacterial (A) and eukaryotic (B) phylogenetic affiliations amongst all four metatranscriptomic libraries prepared from *Pycnopodia helianthoides* tissues. Phylogenetic affilitation was determined by BLASTx analysis against the non-redundant (nr) database at NCBI. Affiliations were normalized by dividing the percentage of reads amongst all reads by the length of contigs assigned to that affilitation.

**Fig 2:**
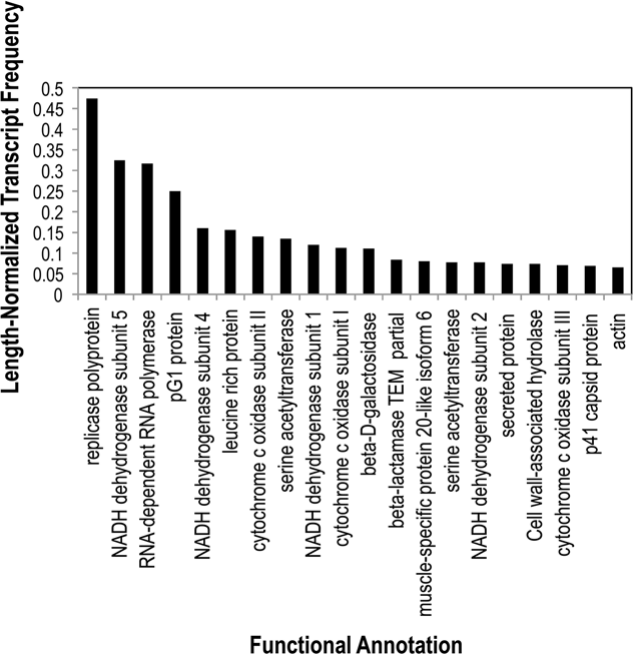
Twenty most highly expressed functional annotations represented in sea star transcriptomes across all 4 libraries. Functional annotations were performed by BLASTx comparison against the non-redundant database at NCBI. Transcript frequencies were normalized by dividing percentage of reads associated with each annotation by contig length.

Amongst bacterial annotations, those associated with *Pseudomonas* and *Vibrio* comprised the greatest proportion (normalized percentage = 3.6 and 3.0), followed by other Gammaproteobacteria, Firmicutes, and Mycobacteria (Fig 1A). Eukaryotic annotations were highest for the Placozoan *Trichoplax adherens* and for the echinoderm *Strongylocentrotus purpuratus*, followed by *Nematostella vectensis* and *Pisaster ochraceus* (Fig 1B). Viral transcripts included genes of ssRNA and ssDNA viruses noted in previous metagenomic surveys [11]. Amongst functionally annotated transcripts, conserved hypothetical, predicted, and domains of unknown function made up the bulk, and proteins involved in replication, citric acid cycle, sulfur metabolism, and aerobic respiration comprised dominant processes across all four libraries (Fig 2). Our observation of a large proportion of bacterial genes in the body wall transcriptomes, particularly those of well-known *r*-selected genera like *Pseudomonas* and *Vibrio*, suggest they may be active constituents of the holobiont, and may rapidly respond to compromised host tissues.

**Fig 3:**
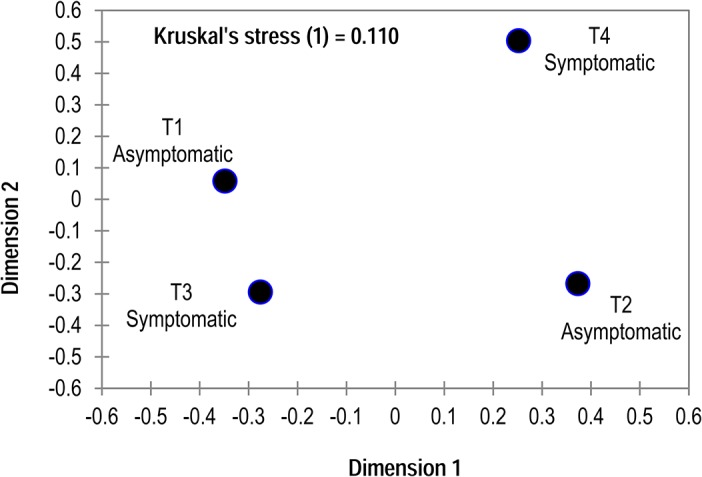
Similarity between overall metatranscriptomic profiles based on Manhattan Distance. Similarity was calculated based on gene-level annotations, and clustered by multidimensional scaling (MDS).

**Fig 4:**
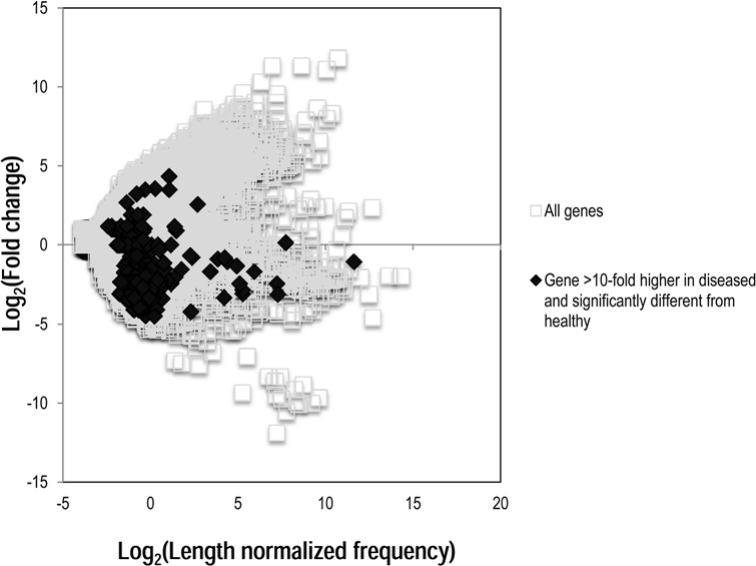
Graphical representation of genes that were significantly over- and under-represented amongst all genes in syptomatic and asymptomatic metatranscriptomes. Fold change was calculated by dividing length-normalized frequency in symptomatic by asymptomatic libraries.

Individual metatranscriptomic libraries were highly variable, with duplicate asymptomatic and symptomatic libraries sharing only 29% and 14% of total annotations, respectively. This variability was higher than between asymptomatic and symptomatic transcripts (30%; i.e. replicate libraries were more variable than between symptomatic and asymptomatic libraries). The strong variability between duplicate transcriptomes influenced the detection of statistical differences between libraries. Clustering of overall transcriptomes by the distribution amongst pathways demonstrated strong heterogeneity between overall metatranscriptomic profiles (Fig 3). We constrained our analysis to transcripts which were > 10 fold higher or lower in abundance in asymptomatic than in symptomatic libraries, and considered transcripts meeting this criterion which also were significantly (Student’s *t*-test, p< 0.05, df = 2) different between tissue states. From here on these are referred to as differentially expressed genes. Transcripts fitting these criteria were predominately low level transcripts that were expressed in both libraries (Fig 4), suggesting that most highly expressed transcripts were either equally present between libraries, or variability in their expression masked their statistical difference between tissue states. The vast majority of genes significantly higher in symptomatic tissues (n = 69) and those significantly lower in symptomatic tissues (n = 332) represented either sequences with no homology to known proteins (40 in symptomatic>asymptomatic, 0.01% of all transcripts; 36 in asymptomatic>symptomatic, 1.03% of all transcripts) or proteins of unknown function (12 in symptomatic>asymptomatic, 0.01% of all transcripts; 86 in asymptomatic>symptomatic; 0.09% of all transcripts). This large number of sequences is in line with metatranscriptomic efforts elsewhere [14], and reflects both the large number of uncultivated microorganisms in marine habitats and dissimilarity between sequenced echinoderm genomes (and expressed sequence tag libraries) and *P*. *helianthoides*.

**Fig 5:**
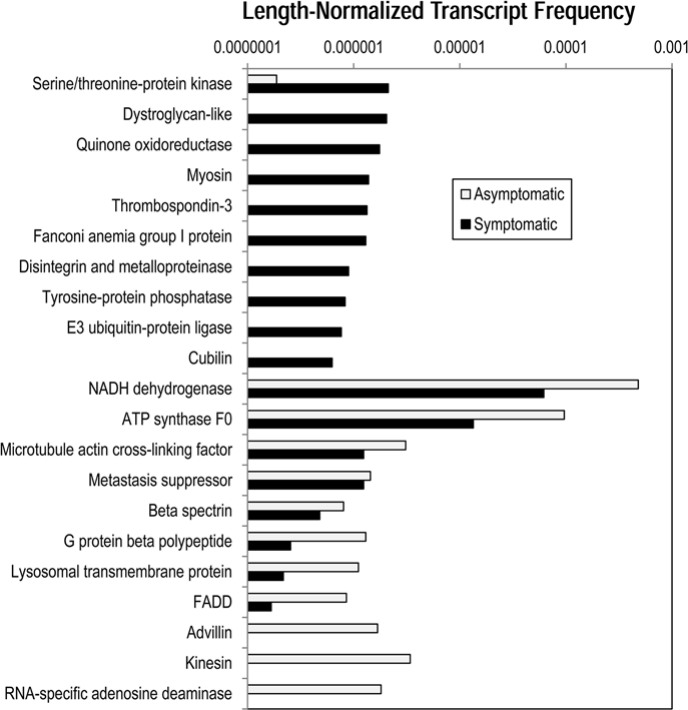
Transcripts occurring at significantly greater frequency in asymptomatic and symtomatic libraries. Only transcripts with functional annotation (vs. hypothetical or unknown function) and putative echinoderm-derived transcripts are indicated. Annotation was based on BLASTx comparison to the non-redundant (nr) database at NCBI.

The overall pattern of differentially expressed genes (i.e. more than 10-fold and statistically significant (p< 0.05, Student’s *t*-test) between asymptomatic and symptomatic tissues) reflects tissue structural changes and disintegration, enhanced protein degradation and autocatalytic protein modification, decreased energy metabolism, and changes in cellular signaling processes (Fig 5). Decreases in expression of several proteins indicate that SSWD may affect the ability of tissues to maintain both cell turgor and cellular morphology. These include β-spectrin, an actin-associated component of the cytoskeleton, microtubule-actin cross linking protein, which functions in stress fiber formation, and advillin, which binds Ca^++^ to actin filaments and has been implicated in cytoskeletal actin regulation. Additionally, enhanced expression of several proteins suggests that SSWD induces decreases in cell adhesion via destruction of matrix architecture and overall matrix remodeling. These include disintegrin, an antagonist of integrin-extracellular matrix protein interactions, and metal efflux pumps and matrix metalloproteinases, which proteolytically degrade matrix components. We also observed elevated dystroglycan transcription, a transmembrane glycoprotein that binds intracellular actin filaments to the extracellular matrix, which may be upregulated as a compensatory response to altered matrix architecture [15]. Taken together, these results indicate that SSWD induces structural changes in both cells and tissues of affected *P*. *helianthoides*, resulting in alterations in the cytoskeleton, extracellular matrix structure, and cell-matrix connections. These multi-level structural changes may explain the tissue sloughing observed in lesions.

**Fig 6:**
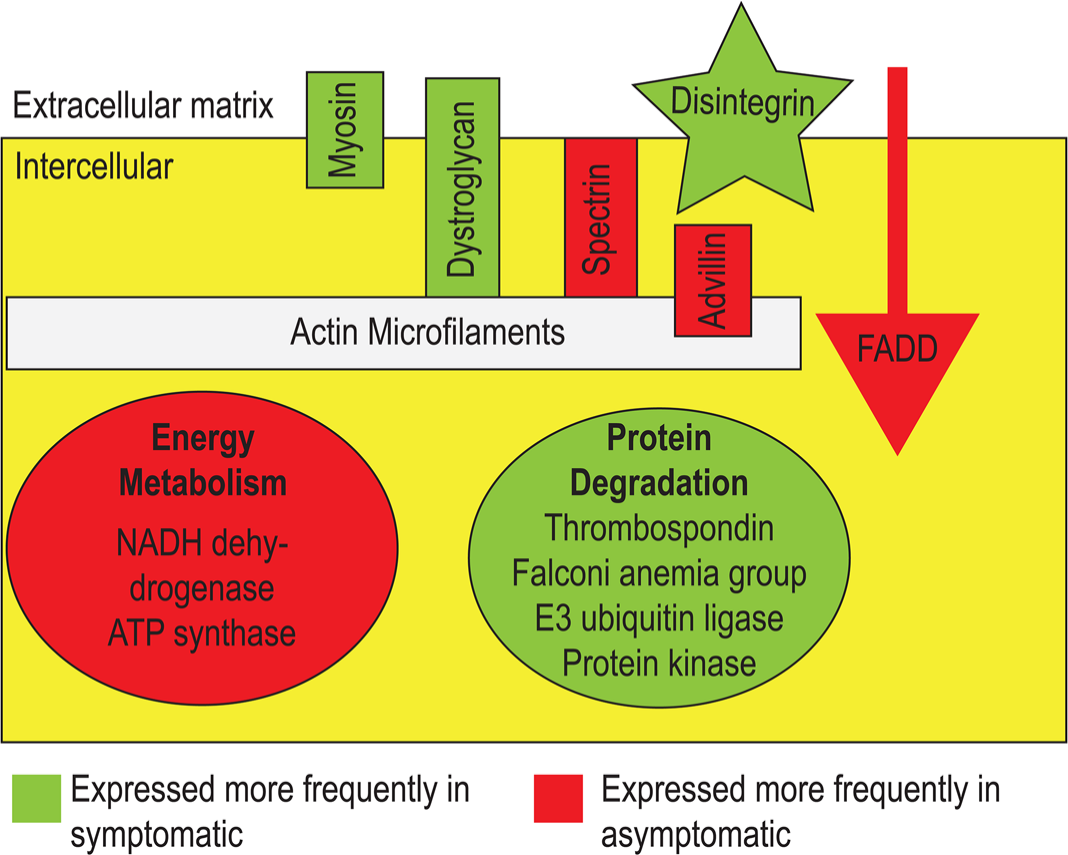
Graphical representation of pathways that were significantly higher in symptomatic or in asymptomatic sea stars.

Alteration in transcriptional levels of signaling pathway transducers is also apparent in response to SSWD. The transcriptional elevation in transmembrane receptor tyrosine kinase and phosphatase, both of which have fundamental roles as regulators of many normal cellular processes, indicates that SSWD alters mediators of kinase signaling cascades within affected cells. This possibly results in downstream transcriptional changes and post-transcriptional protein modifications (Fig 6). Furthermore, decreases in transcription of kinesin motor proteins suggests that SSWD may induce changes in motor-driven vesicle trafficking and in signaling and secretion pathways that rely upon such proteins.

The lower metabolic activity-related gene transcription (i.e. NADH dehydrogenase, ATP synthase) in cells and enhanced transcription of genes involved in protein modification, degradation, and apoptosis induction (thrombospondin, ubiquitin ligase, Falconi-anemia group-like protein, protein kinase and tyrosine protein phosphatase) suggest that SSWD affected tissues undergo significant post-transcriptional changes of proteins in response to the disease, in line with observations of lesion formation and tissue degradation. Limb abscission is a common response of echinoderms to stress, including potentially pathogen invasion [16]. The transcription-level processes leading to autotomy have not been previously described, however they likely involve degradative processes, including the breakdown of extracellular matrices, and possibly the induction of cell death.

The mechanisms by which asteroids experiencing SSWD are affected by SSaDV are not yet fully resolved. This study suggests that SSWD affected body wall tissues of affected asteroids may experience tissue degradation, which is consistent with outward signs of tissue sloughing and lesion formation. Furthermore, induction of apoptotic and protein degradation genes suggest that affected stars may excise or degrade compromised tissues. However, much remains to be learned about the etiology of SSWD, since the large number of bacterial transcripts and greater expression of genes responsible for apoptosis may indicate host response to bacterial holobiont constituents. Further transcriptomic study of immune-specialized cells, e.g. coelomocytes, may provide further clues as to how asteroids respond to disease, and further elucidate the cascade of cellular changes between pathogen exposure and symptom development.

## References

[1] Paine RT (1966) Food web complexity and species diversity. Amer Natur 100: 65–75.

[2] Lessios HA, Cubit JD, Robertson DR, Shulman MJ, Parker MR, Garrity SD et al (1984) Mass mortality of *Diadema antillarum* on the Caribbean Coast of Panama. Coral Reefs 3: 173–182.

[3] Lessios HA (1995) *Diadema antillarum* 10 Years after mass mortality—Still rare, despite help from a competitor. Proc Royal Soc B 259: 331–337.

[4] Lessios HA (2005) *Diadema antillarum* populations in Panama twenty years following mass mortality. Coral Reefs 24: 125–127.

[5] Scheibling RE, Hennigar AW (1997) Recurrent outbreaks of disease in sea urchins *Strongylocentrotus droebachiensis* in Nova Scotia: evidence for a link with large-scale meteorologic and oceanographic events. Mar Ecol Progr Ser 125: 167–173.

[6] Dungan ML, Miller TE, Thomson DA (1982) Catastrophic Decline of a Top Carnivore in the Gulf of California Rocky Inter-Tidal Zone. Science 216: 989–991. 17809070 10.1126/science.216.4549.989

[7] Eckert G, Engle JM, Kushner D. Sea star disease and population declines at the Channel Islands; 1999. pp. 435–441.

[8] Bates AE, Hilton BJ, Harley CDG (2009) Effects of temperature, season and locality on wasting disease in the keystone predatory sea star *Pisaster ochraceus* . Dis Aquat Org 86: 245–251. 10.3354/dao02125 20066959

[9] Zann L, Brodie J, Vuki V (1990) History and dynamics of the crown-of-thorns starfish *Acanthaster planci* (L) in the Suva Area, Fiji. Coral Reefs 9: 135–144.

[10] Pratchett MS (1999) An infectious disease in crown-of-thorns starfish an the Great Barrier Reef. Coral Reefs 18: 272–272.

[11] Hewson I, Button JB, Gudenkauf BM, Miner B, Newton AL, Gaydos JK, et al (2014) Densovirus associated with sea-star wasting disease and mass mortality. Proc Nat Acad Sci USA 111: 17276–17283. 10.1073/pnas.1416625111 PMC4260605 25404293

[12] Gudenkauf BM, Eaglesham JB, Aragundi WM, Hewson I (2014) Discovery of urchin-associated densoviruses (Parvoviridae) in coastal waters of the Big Island, Hawaii. J Gen Virol 95: 652–658. 10.1099/vir.0.060780-0 24362962

[13] Ferguson JC (1996) Madreporite function and fluid volume relationships in sea urchins. Biol Bull 191: 431–440. 29215926 10.2307/1543016

[14] Poretsky RS, Hewson I, Sun SL, Allen AE, Zehr JP, Moran MA, et al (2009) Comparative day/night metatranscriptomic analysis of microbial communities in the North Pacific subtropical gyre. Environ Microbiol 11: 1358–1375. 10.1111/j.1462-2920.2008.01863.x 19207571

[15] Allikian MJ, Hack AA, Mewborn S, Mayer U, McNally EM (2004) Genetic compensation for sarcoglycan loss by integrin alpha 7 beta 1 in muscle. J Cell Sci 117: 3821–3830. 15252120 10.1242/jcs.01234

[16] Pincebourde S, Sanford E, Helmuth B (2013) Surviral and arm abscission are linked to regional heterothermy in an intertidal sea star. J Exper Biol 216: 2183–2191. 10.1242/jeb.083881 23720798

